# Correlation of Motor-Task Related Brain Activation Changes With Motor Performance Changes Induced by Constraint-Induced Therapy After Stroke

**DOI:** 10.1177/15459683261464076

**Published:** 2026-07-21

**Authors:** George F. Wittenberg, Xiaoyan Leng, Andrew J. Butler, Krishnankutty Sathian

**Affiliations:** 1 Wake Forest University SOM, Department of Neurology, Program in Rehabilitation, Winston-Salem, NC, USA; 2 University of Pittsburgh SOM, Departments of Neurology, Physical Medicine & Rehabilitation, and Bioengineering, Pittsburgh, PA; 3 TECH-GRECC, HERL, VA Pittsburgh Healthcare System, Pittsburgh, PA, USA; 4 Wake Forest University SOM, Departments of Biostatistics and Data Science, Public Health Sciences, Winston-Salem, NC, USA; 5 Emory University, School of Medicine, Department of Rehabilitation Medicine, Atlanta, GA, USA; 6 University of Alabama, School of Health Professions and Department of Physical Therapy, Birmingham, AL, USA; 7 Emory University, School of Medicine, Department of Neurology, Atlanta, GA, USA; 8 Atlanta VAMC Rehabilitation R&D Center of Excellence in Rehabilitation of Aging Veterans with Vision Loss, Decatur, GA; 9 PennState College of Medicine, Neuroscience Institute, Departments of Neurology, Neuroscience and Experimental Therapeutics, Hershey, PA

**Keywords:** stroke, constraint-induced movement therapy, functional MRI, motor recovery, cortical plasticity

## Abstract

**Background:**

Steven Wolf made an exceptional effort to design, organize, and conduct a study of the biology of constraint induced movement therapy (CIMT) but only the transcranial magnetic stimulation results have been published previously.

**Objective:**

To evaluate changes in motor-task related brain activation and their relationship to functional recovery in participants with stroke undergoing CIMT during the subacute and chronic periods.

**Methods:**

Participants with hemiparetic stroke (n = 42) underwent fMRI of a hand task at baseline, immediately after a 2-week intervention or waiting period, and 4 months later, with effort or rate controlled at each timepoint. Functional outcome was assessed via the Wolf Motor Function Test (WMFT). Functional images were analyzed at the individual and group level.

**Results:**

Significant improvement in WMFT occurred in the intervention groups. Group-level changes in task-related activation over time were not significant. However, across all participants, increased WMFT task speed correlated significantly with increased ipsilesional M1 activation. No significant difference between activation changes were noted between any groups.

**Conclusions:**

Despite meaningful functional gains, longitudinal changes in cortical activation were limited when controlled for rate or effort, yet individual recovery was significantly associated with preserved or increased activation in the affected primary motor cortex. These results suggest that post-stroke motor recovery may reflect changes in how effort to move activates the stroke-affected motor cortex and more distal changes in response to motor cortical activation. Multi-site fMRI in stroke rehabilitation trials remains challenging, with strong control and monitoring of task performance needed.

## Introduction

*This paper, mothballed for several years, describes part of a substudy of EXCITE. The EXCITE study was a landmark, multisite, randomized controlled trial designed to evaluate Constraint-Induced Movement Therapy (CIMT) as a method of improving upper extremity motor function in stroke-affected people 3-9 months after stroke.*^1^ *It demonstrated that CIMT significantly improved arm motor function and functional use, with a larger effect if the treatment was earlier but with long-term equalization of outcomes.*^2^ *I (GFW) was involved in this study soon after I arrived at Wake Forest in 2000, where EXCITE had already begun. David Good organized us to submit a grant application to follow brain changes expected to be induced by CIMT over time and correlate such changes with recovery. As I recall, the application had to be resubmitted before it was funding, losing valuable time during which we could have recruited EXCITE participants. Steve Wolf had a very active and direct role in organizing and completing the project. He visited Wake Forest and the Ohio State University to ensure that procedures were being done correctly (as he did at Emory as well) and introduced us to the* close bipolar montage *for EMG recording, something I have used ever since. But despite best intentions and efforts of a large team at 3 institutions, the MRI results have not been published before, although the TMS ones have.*^3,4^ *The paper that follows was completed from a draft of about 10 years ago. Analyses had been done over the years and the exact statistical procedures used for some have been lost, although there were good records of the imaging analyses, thanks to Allison Fowlkes. I also found a six-page set of notes on the “FICIT Summit Meeting” held 27 March 2006. These were helpful and include multiple perspectives on the data. I believe Steve was the author but may not have been the notetaker.*

The effect of recovery and rehabilitation processes on functional organization of motor activity has been an active area of functional imaging research^5-7^ with some consensus results about the role of the unaffected hemisphere in recovery.^8-11^ There is a normalization of activation in patients that recover well, although brain activity related to movement may involve more activation of both normally active and additional motor areas in both hemispheres. Interest remains in using transcranial magnetic stimulation (TMS) and MRI as predictive measures of response to therapy.^12,13^

The subacute period after stroke is a time of continued recovery that may also represent a period of increased response to interventions.^14^ The EXCITE trial was designed to measure recovery during the later part of that period in response to constraint-induced therapy (CIMT), a method that remains one of the principal research methods for stroke trials (e.g., Schlaug et al^15^). It has also been demonstrated to influence motor maps as measured by transcranial magnetic stimulation^16,17^ and MRI^18,19^ in small studies using each modality alone, and in a single subject report using both.^20^ The MRI results reported here are from data acquired during the EXCITE trial and for a year after its conclusion, using the same methodology in an additional cohort of participants. About 20 years have passed, but there is more interest in negative trials and continued interest in the scientific questions. Of course, caution needs to be applied to the results and to consider them exploratory, and even the basic negative result of non-significant change of task-related activation over time could be construed to be due to a lack of sensitivity of older fMRI methods.

One innovation in this study, which was carried over from prior work,^21^ was to address the problem of variable task performance between sessions. Because rate of movement influences brain activation with fMRI,^22^ we sought to keep rate constant from session to session. However, if the effort required to move at a particular rate changes, this may change brain activation as well, particularly in structures related to motor executive function.^23^ We therefore pursued a dual strategy: constant rate (1Hz) tasks during some scans and tasks at a percentage (70%) of the maximum rate possible at the time of each scan during others. In the end, that did not make any significant difference in the results but was an important control.

### Subjects and Methods

**Figure 1.**
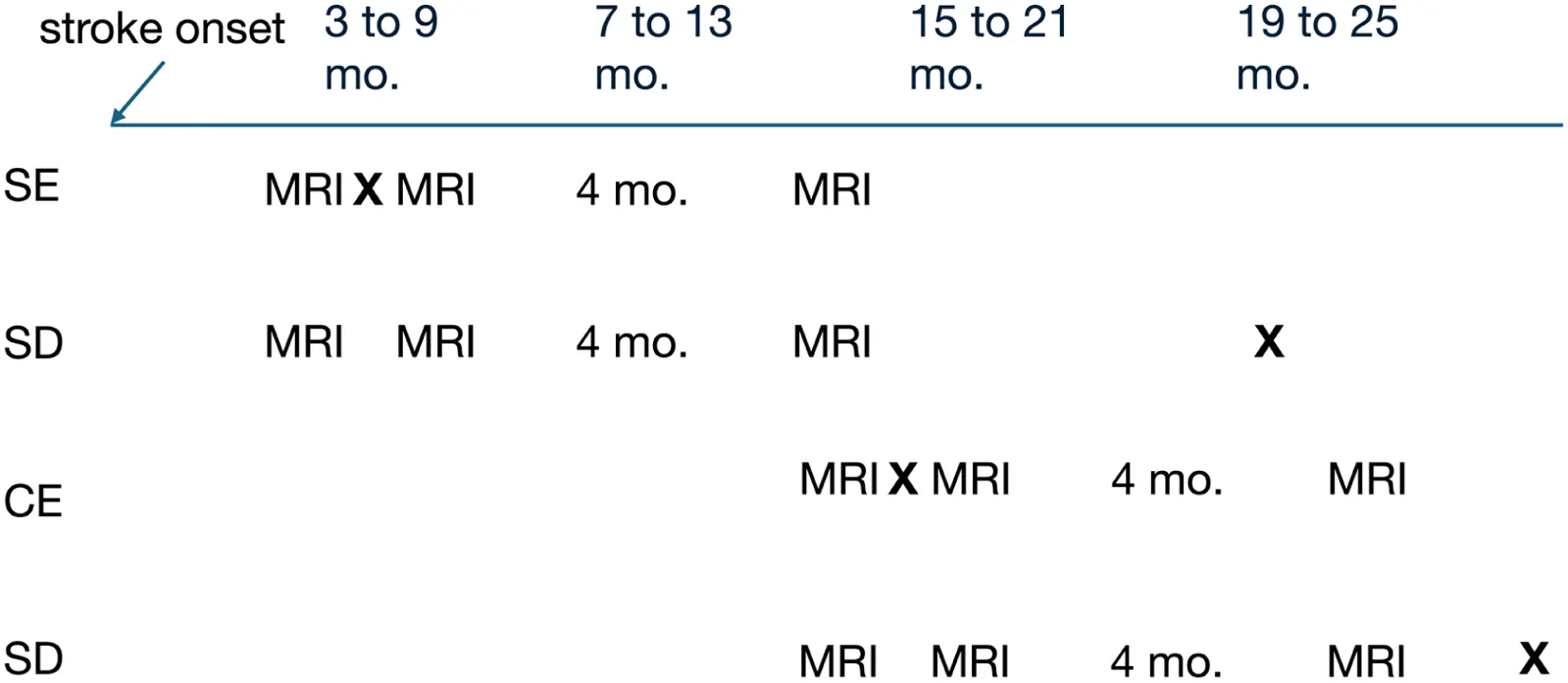
Timeline of interventions and MRI. The 4 groups are: SE – Subacute Experimental, SD – Subacute Delayed, CE – Chronic Experimental, and SD – Subacute Delayed. Time is indicated by the line at the top, with approximate time after stroke onset. “**X”** indicates the time of the 2-week intervention, and “MRI” the time of the MRI.

The results reported here include the subacute subjects reported on in Sawaki et al^3^ and chronic subjects in Sawaki et al.^4^ A brief synopsis of methods follows:

**Subjects:** This study was layered upon the EXCITE study, with 17 subjects initially recruited exclusively from within that study, at 3 participating sites. After the conclusion of EXCITE enrollment, an additional 30 subjects were recruited, with identical criteria and treatment. EXCITE involved therapy during a subacute period (3-9 months post stroke) and randomly assigned immediate treatment – subacute experimental (SE) – or treatment delayed by 4 months – subacute delayed (SD). Two chronic groups were added for comparison to previous studies of CIMT, with 1 chronic group receiving immediate treatment – chronic experimental (CE) and the other delayed (CD) (Figure 1). All subjects had MRI scans at baseline, at 2 weeks, and at 4 months, with the delayed groups serving as controls for the effects of time and usual care. Each individual participant gave informed consent. The protocol was approved by the Institutional Review Boards at each participating site (Wake Forest University, Emory University and The Ohio State University).

#### Study Design

*Active Treatment Groups*: Each subject in a treatment group (subacute or chronic) participated in 10 consecutive weekdays of intensive upper extremity therapy during which time they donned a padded mitt covering the less affected hand. Treatment focused on the affected upper extremity, with shaping of movements to accomplish functional goals. Tasks involved grasp, manipulation, and release of objects.

*Control Group*: The control (delayed treatment) group continued with their usual and customary care but had the same measures as the active treatment groups over the same period.

##### Outcome Measures

*Wolf Motor Function Test (WMFT)*: The WMFT^24^ was the primary clinical outcome measure and was performed by blinded evaluators at all sites. This test has established reliability, and validity and has also been used extensively to evaluate upper extremity motor function in CIMT trials. It encompasses a battery of 15 time-based and 2 force-based tasks.

### fMRI Methods

Brain activation was measured for voluntary movement at the metacarpophalangeal (MCP) joints. While wearing a splint designed to position the wrist in a neutral position and to limit the amplitude of movement, the participants repeatedly extended and flexed the fingers of one hand simultaneously at the MCP joints. The splint was specifically designed to position the hand at an optimal biomechanical advantage to allow for flexion and extension of the MCPs. Movement was paced by an auditory signal at the rate of 1 Hz or at 70% of the maximum rate the participant could achieve on the day of the scan. To measure finger movements and detect mirror movements, a fiber optic bending transducer was secured at the dorsal base of the second nail bed of each hand (S 720 Shape Sensor, Measurand Inc, Fredericton, NB, Canada). The movement amplitude was set to the available active range of motion. This fixed range of motion was determined at the first scanning session for each participant and was applied without changes at all subsequent scanning sessions. The task was performed on both sides and at 2 rates: (1) right hand 1 Hz, (2) right hand 70% max. rate, (3) left hand 1 Hz, and (4) left hand 70% max. For each task, 30 second periods of task alternating with 30 second periods of rest, with 4 rest periods and 3 task periods per run (BABABAB design). Each subject was asked to perform 2 runs of each task type.

This task of finger flexion/extension was chosen because it would be possible for any participant who had the minimal motor criteria for entry into the study. A rate of 70% maximum rate was able to be performed without fatigue in stroke patients throughout the scan, with minimal head movement that often accompanies difficult tasks. Prior to each functional run, volunteers were instructed to keep their eyes closed. All participants were trained prior to each scanning session to accurately and consistently perform the unilateral hand motor task. A research investigator, standing adjacent to the scanner, visually monitored hand motor performance and possible unintended movements, including mirror movements of the contralateral hand. Subjects were verbally encouraged to continue moving or to maintain the task rate, if necessary, but otherwise distractions were minimized.

#### Imaging Protocol

In order to standardize data acquisition formats, imaging at each site was performed on 1.5 Tesla scanners using a standard acquisition technique on Signa Echo- or Twin-Speed systems (GE, Minneapolis, MN) at Wake Forest University and Ohio State University and on the Intera system (Philips, Eindhoven, Netherlands) at Emory University which had been shown to produce comparable results. Imaging consisted of a routine sagittal T1-weighted localizer followed by a high-resolution axial T1-weighted acquisition of the entire brain. The high-resolution images were used both for anatomic co-registration with TMS, as well as for spatial normalization of the data sets to a standard atlas. Functional imaging, performed with EPI sequences, involved standard single-shot gradient echo dynamic acquisition with FOV = 24, 64 × 64 matrix, 28 slices, 5 mm thickness, no skip, TR = 3.0 seconds, and TE = 40 ms. Raw images were reconstructed off-line on a remote workstation at each site and sent to Wake Forest University for further analysis.

### fMRI Analysis

Statistical parametric maps (SPMs) were generated using FSL.^25^ Data was normalized using the high resolution T1 image. The FSL tools FEAT v 5.4 was used for the first level and higher-level analysis with FLAME. The T1-weighted images were normalized to a standard template in Montreal Neurological Institute (MNI) coordinate space. The functional data sets were motion corrected (intra-run realignment) using the first image as the reference. Runs with movement magnitude greater than 2 mm were rejected. The functional data was normalized to the T1 image and smoothed with a 4 mm kernel. All images from people with left-sided brain lesions were flipped so analysis was as if all strokes were right-sided and the left hand was the more affected one.

### ROI Analysis

Regions of interest were derived from the atlases provided with WFU_PickAtlas.^26^ There were selected and expanded or contracted to cover the primary motor cortex (M1), primary sensory cortex (S1), premotor area (PMA), supplementary motor area (SMA), inferior and superior parietal cortices (IP, SP), and cerebellum on each side of the standard template image. The mean beta, representing the magnitude of activation of each region, was calculated using the FEAT function of FSL.

### Statistical Analysis

Longitudinal analysis of whole brain task-related activation was performed in FSL.^27^ First-level analysis was performed for each scan and then second level analysis included covariates of time, with random-effects. Significance was set at *P* < .05 with FSL’s correction for false discovery rate.

Scatterplots, ANOVA, and linear regression were performed using GraphPad Prism version 11.0.1 for Macintosh (GraphPad Software, Boston, Massachusetts USA.)

### Mothballing Issues

The FSL analysis was done close in time to the completion of the trial and conclusions reflect the results, with no attempt to reanalyze historical data. This has advantages of reducing bias, but application of more recent methods could have resulted in different results. Analysis of ROI-based correlations with functional outcomes are more likely to be valid, as they are based on means of voxel signals.

## Results

**Figure 2.**
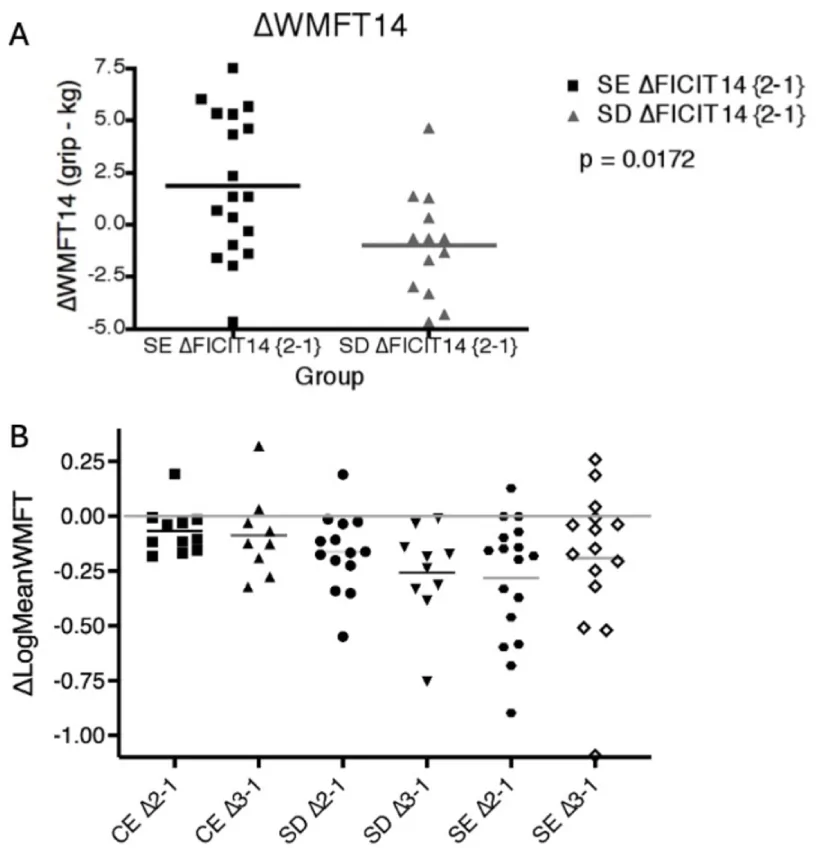
Selected change in WMFT measures for the 2 largest groups: (A) Grip Strength (WMFT 14) change over the intervention (referred to as 2-1; Note that grip is given in kg while the SI unit of force is newtons(N). To convert to N, multiply by 9.8.) Mean grip strength increased in the subacute experimental group, while it decreased in the subacute delayed group, which received no therapy during this time interval. (B) Change in log(mean WMFT time) for the 3 largest groups. Means are indicated by the horizontal lines. (B) Changes over the intervention interval (2-1) or entire study interval (3-1) are shown. All comparisons indicate faster times for the group mean over time. With a planned comparison of only the 2-1 intervals, the difference between CE and SE was significant (*P* = .03, Tukey’s multiple comparison’s test.).

Subject **characteristics** for the subacute groups were described in Sawaki et al.^3^ Briefly, there were 46 participants initially, with 10 withdrawals before final evaluations and 1 withdrawal due to declination of active treatment. There were 13 chronic stroke participants, of whom 11 received CIMT immediately and 33 subacute participants, of whom 16 received CIMT immediately. The remainder of the participants in each group received CIMT after a delay but did not have MRI during that period. The groups were: (1) subacute experimental (SE), (2) subacute delayed (SD), (3) chronic experimental (CE), and (4) chronic delayed (CD.) Because there were only 2 participants in the (CD) group that group was dropped from analysis. The 3 remaining groups were well-matched in terms of age and motor ability and distributed evenly over the 3 institutions involved, although Wake Forest had only 1 chronic participant. Specifically, at baseline neither clinical variables (WMFT and FM) in the SE group were significantly different from either other group at the *P* < .05 level. The clinical outcomes of all groups demonstrated improvements in mean log WMFT time with a significant effect of group and time (F (2, 39) = 3.513, *P* = .04, Figure 2).

## Motor Activation Over Time

**Figure 3.**
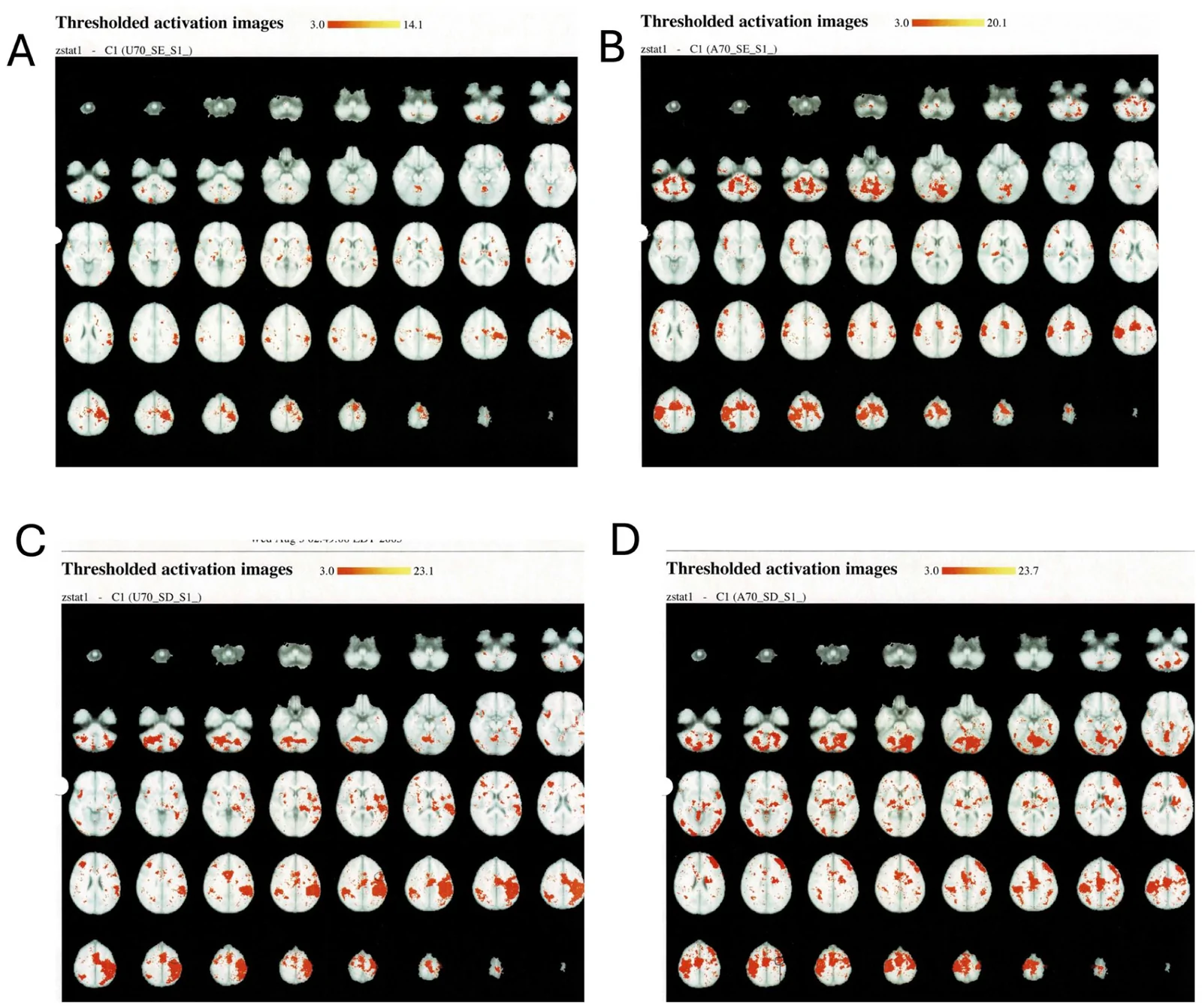
FSL output for the contrast of task vs rest for the subacute groups moving the fingers at 70% maximal frequency prior to the intervention. The right (affected) side of the brain is on the left. (A) SE group, unaffected side task. (B) SE group, affected side task. (C) SD group, unaffected side task. (D) SD group, affected side task.

Brain activation images were created for each participant, at each time point, and for each motor condition and analyzed as a group. Focusing on the largest, SE group: Brain activation related to the task on the unaffected side showed expected significant clusters, including primary motor cortex and supplementary motor area, cerebellar vermis and inferior hemispheres (Figure 3). The affected side activation including all these regions, as well as the unaffected hemisphere dorsal and ventral premotor regions, and more voxels in the cerebellum bilaterally. Analysis of change across time was performed separately for the two task rates, because in the case of 70% maximum rate, effort is kept constant across time, while in the 1 Hz case, rate is kept constant. This SE group showed almost no voxels with significant *change* in brain activation from the first to either second or third timepoint. There was only a small right (contralesional) cluster of cerebellar voxels that appeared to have increased over time in this group. The SD group, which received no therapy during the study period, had a similar pattern of activation at the outset (Figure 3) and over the course of the study. There were no significant changes over time. The CE group had overall poorer activation images but also had lack of significant change over time.

## Imaging Changes and Functional Ability Changes

**Figure 4.**
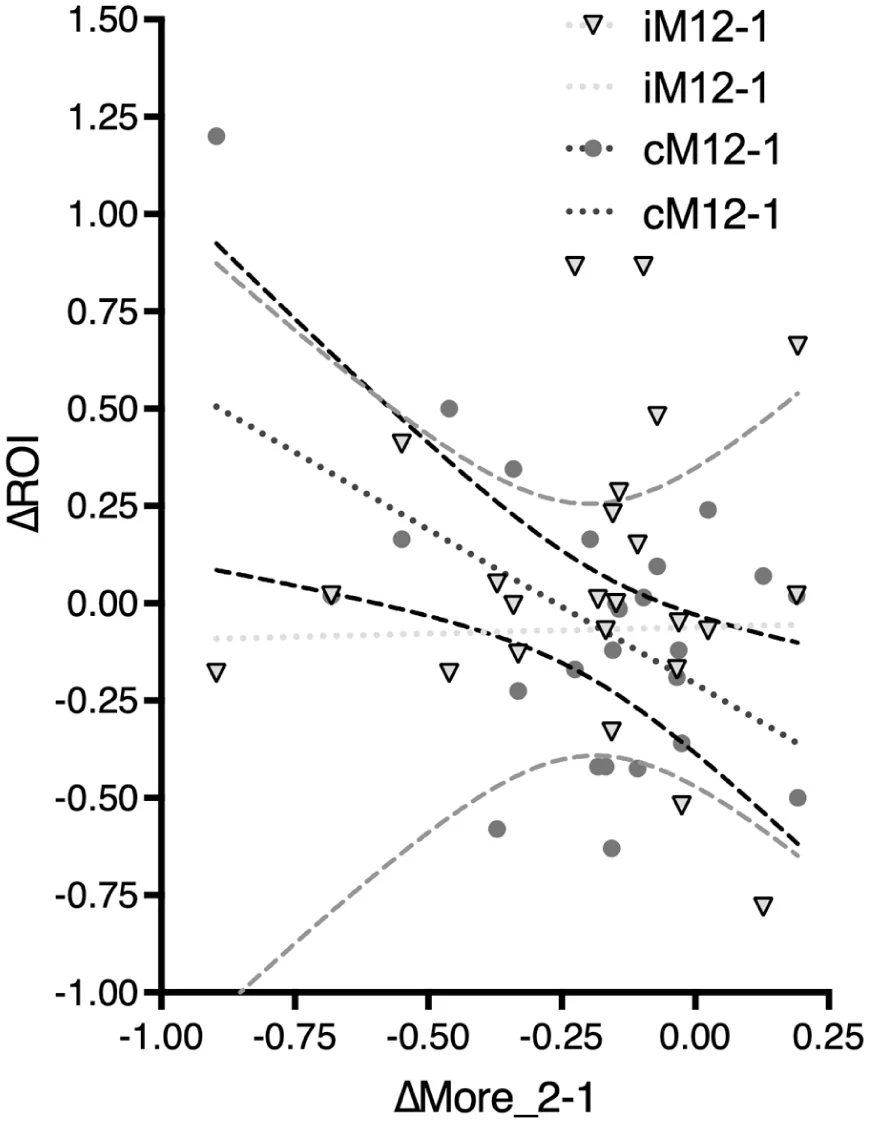
All group correlation between WMFT log mean time and change over the intervention period in M1 activation ipsilateral to the affected hand (iM1) or contralateral to the affected hand (cM1) with 70% effort of the affected hand. Regression lines with 95% confidence intervals are indicated. The correlation line for the contralateral (affected hemisphere) was significantly different from zero (*P* = .008).

With little *mean* change in any group for brain activation, we then examined whether any brain activation changes correlated with change in Wolf Motor Function Test (WMFT) scores. At the first step of analysis, the WMFT time was correlated with task-related brain activation for each participant at each timepoint. The group pattern of correlation was noisy but consistently included ipsilesional premotor and sensorimotor cortices. Higher WMFT time was correlated with more activation, although there was variation in significantly correlated voxels across time. Across all groups, the change in Wolf Motor Function Test time over the intervention interval (S2-S1) was negatively correlated (slope −0.794 ± 0.272 S.E.) with change in affected hemisphere M1 mean activation (beta) for the 70% effort task and this correlation was significant but small (r^2^ = 0.271, F = 8.547, *P* = .0076.) This would imply that more improvement in function was associated with an increase (or preservation) of activation. Other regions: Change in the unaffected hemisphere M1 was not significant (Figure 4). No other regions of interest showed significant correlations with WMFT time.

Because WMFT item 14 (grip strength) is different than most others, its correlations were analyzed separately and in an exploratory fashion. For the SE group the only significant correlation was negative: between change in grip between S1 and S2 and change in contralesional superior parietal area activation (−0.62 slope, *P* = 0.04, n = 11.). The same held true in the SD group (−0.78 slope, *P* = 0.03, n = 7) There were no significant relationships for the S3 interval comparisons.

## Discussion

This study examined the changes in distal upper extremity task-related brain activation and response to treatment at 2 different times after an intervention and in 2 groups differing in their time after stroke. The functional outcomes were consistent with the parent EXCITE study. The principal MRI results were that this activation did not significantly change in any group, whether or not they received CIMT. That is, there were no significant effects of intervention or time after stroke. There was a significant relationship between changes in motor functional measures and changes in bilateral M1 activation and contralesional superior parietal activation.

Functional outcomes in this supplemented subset of the EXCITE trial could be compared to the overall EXCITE results.^2^ In that study WMFT time was significantly reduced after treatment in the subacute group – from 30 seconds to 20 seconds and sustained after a year at 21 seconds, while the chronic group also had a significant but smaller reduction in WMFT time from 27 to 22 seconds and barely sustained at 25 seconds after a year. For the subset of EXCITE participants in this study, the pattern was similar, but the chronic group had a slightly smaller change. The subset of participants in this study thus were similar to those in the parent study and reflect greater responsiveness to the intervention when provided earlier.

Mean M1 activation for 1 Hz finger movement appeared to decrease after CIMT for subacute patients, which is similar to prior findings^28^ but those with an increase (or more preservation) of M1 activation had the best recovery. This apparent paradox may stem from altered spinal cord responsiveness to upper motor neuron activation. The TMS studies from the same group of participants showed that CIMT led to an expansion of motor maps^3,29^ more so in chronic than subacute participants,

Post-stroke motor recovery due to unilateral task-practice may therefore reflect subtle or no changes in how a simple motor behavior activates multiple motor-related cortical areas in both hemispheres. All EXCITE groups showed expected activation of the motor network with gross hand movements that were carefully controlled for frequency and effort. Despite improvements in motor function in the groups with the intervention, no group had a statistically significant change in motor task-related brain activation over time, when either task effort or rate were controlled. These results are at variance with a prior study^30^ but the time after stroke was much later in those participants and demonstrate some advantages of measuring the effects of an intervention in a more stable state.

Improvement in WMFT times was correlated with increased activation of M1 on both affected and unaffected side with affected side movement, suggesting that functional use of the impaired arm was related to increased activation or at least *preservation* of task-related activation in the M1 in both hemispheres. There was no group difference in these relationships, meaning that timing of the intervention had little effect on the relationship of activation change with change in function. This discrepancy between a group change in brain activation and a correlation between functional and imaging outcomes may reflect the variability in responsiveness to the intervention, or a fundamental lack of change in brain activation for well-controlled tasks related to time or intervention in the subacute period.

Decreased activation of the contralesional superior parietal area was correlated with improved grip strength, suggesting that recovery of force production had a different recovery mechanism than for rapid, repetitive movement. The superior parietal result is suggestive of the role of this area in stroke recovery, which has been found in other studies.^31-33^ The overall picture was one of individual changes in task-related activation related to functional gains but not to experiencing the CIMT intervention itself. This contrasts with the TMS results, which have shown the opposite pattern.

## Conclusion

This study found that motor task-related brain activation remained largely unchanged over time following stroke, regardless of intervention timing, though improvements in motor function correlated with preserved bilateral M1 activation and reduced contralesional superior parietal activation. These findings suggest that CIMT may help protect existing motor circuitry from degradation due to learned non-use, rather than fundamentally altering it.
